# Supplementary material for: Vitamin D metabolites and the gut microbiome in older men
Source: Nat Commun. 2020 Nov 26;11:5997. doi: 10.1038/s41467-020-19793-8 (PMC7693238; doi:10.1038/s41467-020-19793-8)
Supplement: Supplementary file 1 — Supplementary Information [file 41467_2020_19793_MOESM1_ESM.pdf]

Supplementary Information

Vitamin D Metabolites and the Gut Microbiome in Older Men

Robert Thomas and Lingjing Jiang et al.

Supplementary Table 1. List of All Primers Used for Microbial DNA sequencing

The 16Sv4 rRNA was amplified using the 515F and 806R primers:

Read1: TATGGTAATTGTGTGCCAGCMGCCGCGGTAA
Index: ATTAGAWACCCBDGTAGTCCGGCTGACTGACT
Read2: AGTCAGTCAGCCGGACTACHVGGGTWTCTAAT

Supplementary Table 2. Solvents, Gradients, Flows and Switching Schemes for 25(OH)D Method.

Table 1: Gradients, flows and solvents for the 25(OH)D method. Solvents coupled to the two Nexera XR LC-20ADxr (gradient pumps) were water containing 0.1% formic acid and pure methanol respectively. The solvents coupled to **LC-20AD** (loading pump) were a mixture of 60:40 *(v/v)* water:methanol and pure methanol. The gradient pumps generated flow over the analytical column in the loading phase and over loading and analytical column in series in the elution phase.

<table>
<tr><th rowspan="2">Time (min)</th><th colspan="2">2x Nexera XR LC-20ADxr</th><th colspan="2">LC-20AD</th><th rowspan="2">Valves</th></tr>
<tr><th>Methanol</th><th>Flow</th><th>Solvent</th><th>Flow</th></tr>
<tr><td>0.00</td><td rowspan="6">Isocratic 79%</td><td rowspan="12">0.75 mL/min</td><td rowspan="6">60:40 (v/v) water:methanol</td><td rowspan="6">0.6 mL/min</td><td rowspan="2">Loading</td></tr>
<tr><td>0.70</td></tr>
<tr><td>0.70</td><td rowspan="2">Elution</td></tr>
<tr><td>1.40</td></tr>
<tr><td>1.40</td><td rowspan="8">Loading</td></tr>
<tr><td>2.90</td></tr>
<tr><td>2.90</td><td rowspan="2">Isocratic 100%</td><td rowspan="4">methanol</td><td rowspan="4">1.0 mL/min</td></tr>
<tr><td>4.50</td></tr>
<tr><td>4.50</td><td rowspan="4">Isocratic 79%</td></tr>
<tr><td>5.20</td></tr>
<tr><td>5.20</td><td rowspan="2">60:40 (v/v) water:methanol</td><td rowspan="2">0.6 mL/min</td></tr>
<tr><td>6.80</td></tr>
</table>

Supplementary Table 3. Mass Transition Settings

	Q1 *(m/z)*	Q3 *(m/z)*	Mode	DT[a] (msec)	DP[b] (V)	CE[c] (V)	CXP[d] (V)
**25(OH)D_3**	383.2	211.1	APCI+	80	90	31	14
**25(OH)D_3**	383.2	229.1	APCI+	80	90	20	12
**25(OH)D_2**	395.2	211.1	APCI+	80	95	31	14
**25(OH)D_2**	395.2	269.3	APCI+	80	95	24	13
***d6*-25(OH)D_3**	389.4	211.1	APCI+	80	90	31	14

[a]Dwell time, [b]Declustering Potential, [c]Collision Energy, [d]Collision Cell Exit Potential

Supplementary Table 4. SAS Code used to create initial MrOS participant data set that was merged with the publicly available microbial sequencing data deposited in the European Bioinformatics Institute (EBI), accession number ERP 107984.

```
LIBNAME library "C:\MrOS\data\FORMATS" ACCESS=READONLY;
libname v4 "C:\MrOS\data\Visit4Data\V4AUG19";
libname v1 "C:\MrOS\data\V1FEB14";
libname vd "C:\MrOS\data\Visit4Data\OH4AUG17";
libname M4 "C:\MrOS\data\Visit4Data\M4AUG16";
libname F4 "C:\MrOS\data\Visit4Data\F4NOV18";
libname mf4 "C:\MrOS\data\Visit4Data\MF4AUG16";

data v1race;
set v1.v1feb14;
keep id gierace;
run;
data v4covariates;
set v4.v4aug19;
keep id V4AGE1 MIDATA HWWGT HWBMI HWHGT site PASCORE TURSMOKE TUDRAMT
qlcomp;
run;
data m4covariates;
set m4.m4aug16;
keep id M1ADEPR M1PROBI M1VITMND M1ANTIB M1PPUMP M1STATIN ;
run;
data mfmeds;
set mf4.mf4aug16 (keep=id M1FEXOFE
M1TERFEN
M1BROMPH
M1CHLORR
M1DIPHHY
M1PROMET
M1DESLOR
M1LORATA
M1CETIRI
M1CLEMAS
M1HYDROY
M1LEVOCE
M1CARBIN
M1DEXBRO
M1DEXCHL
M1MAGCIT
M1LACTUL
M1BISACO
M1MAGSUL
M1SENNA
M1POLYET
M1DOCOSS
M1DOCOSC
M1PSYLLI
M1POLYCA
M1MAGHYD
M1METHCE
M1METFOR);
run;
data diet;
```

```
set f4.f4feb18
(keep=id DTVITD DTSPVITD DTCALC DTSPCALC
DTGMCCER
DTGMBAGL
DTGMBISC
DTGMSSNK
DTGMCRCK
DTGMBANA
DTGRANSV
DTGMRICE
DTGMBEAN
DTGMPOTA
DTGMSPOT);
run;
data OHVD;
set vd.OH4AUG17 (keep=id OHSEAS OHV1D3 OHV24D3 OHV1D2 OHVD2 OHVD3 OHVDTOT
OHV1DTOT);
run;
data all;
merge v1race v4covariates m4covariates mfmeds diet OHVD;
by id;
if MIdata=1;
run;
data allnew;
set all;
IF (M1FEXOFE OR M1TERFEN OR M1BROMPH OR M1CHLORR OR M1DIPHHY OR M1PROMET
OR M1DESLOR OR M1LORATA OR M1CETIRI OR M1CLEMAS OR M1HYDROY OR M1LEVOCE
OR M1CARBIN OR M1DEXBRO OR M1DEXCHL) THEN Antihistamine=1;
ELSE Antihistamine=0;
IF (M1MAGCIT OR M1LACTUL OR M1BISACO OR M1MAGSUL OR M1SENNA
OR M1POLYET OR M1DOCOSS OR M1DOCOSC OR M1PSYLLI OR M1POLYCA OR M1MAGHYD OR
M1METHCE) THEN Laxative=1;
ELSE Laxative=0;
/*****To get dietary VD intake and calculate dietary resistance starch
***************/;
DTGMCCER_rs=DTGMCCER*1.52/100;
DTGMBAGL_rs=DTGMBAGL*1/100;
DTGMBISC_rs=DTGMBISC*1/100;
DTGMSSNK_rs=DTGMSSNK*1.7/100;
DTGMCRCK_rs=DTGMCRCK*1.06/100;
DTGMBANA_rs=DTGMBANA*4/100;
DTGMRICE_rs=DTGMRICE*1.4/100;
DTGMBEAN_rs=DTGMBEAN*1.9/100;
DTGMPOTA_rs=DTGMPOTA*1/100;
DTGMSPOT_rs=DTGMSPOT*0.7/100;
Rstarch_total=DTGMCCER_rs+DTGMBAGL_rs+DTGMBISC_rs+DTGMSSNK_rs+
DTGMCRCK_rs+DTGMBANA_rs+DTGMRICE_rs+DTGMBEAN_rs+DTGMPOTA_rs+DTGMSPOT_rs;
if rstarch_total < 5 then rstarch_c=0;
if rstarch_total >=5 then rstarch_c=1;
totalcalc=DTCALC+DTSPCALC;
totalvitD=DTVITD+DTSPVITD;
if tudramt >.Z then Drink=tudramt;
if tudramt <.Z then Drink=0;
run;
```
