# Supplementary material for: Vitamin D metabolites and the gut microbiome in older men
Source: Nat Commun. 2020 Nov 26;11:5997. doi: 10.1038/s41467-020-19793-8 (PMC7693238; doi:10.1038/s41467-020-19793-8)
Supplement: Supplementary file 3 — Reporting Summary [file 41467_2020_19793_MOESM3_ESM.pdf]

nature research

Corresponding author(s): Dr. Deborah Kado

Last updated by author(s): Oct 12, 2020

Reporting Summary

Nature Research wishes to improve the reproducibility of the work that we publish. This form provides structure for consistency and transparency in reporting. For further information on Nature Research policies, see our Editorial Policies and the Editorial Policy Checklist.

Statistics

For all statistical analyses, confirm that the following items are present in the figure legend, table legend, main text, or Methods section.

n/a	Confirmed	
☐	☒	The exact sample size (n) for each experimental group/condition, given as a discrete number and unit of measurement
☐	☒	A statement on whether measurements were taken from distinct samples or whether the same sample was measured repeatedly
☐	☒	The statistical test(s) used AND whether they are one- or two-sided<br>*Only common tests should be described solely by name; describe more complex techniques in the Methods section.*
☐	☒	A description of all covariates tested
☐	☒	A description of any assumptions or corrections, such as tests of normality and adjustment for multiple comparisons
☐	☒	A full description of the statistical parameters including central tendency (e.g. means) or other basic estimates (e.g. regression coefficient) AND variation (e.g. standard deviation) or associated estimates of uncertainty (e.g. confidence intervals)
☐	☒	For null hypothesis testing, the test statistic (e.g. F, t, r) with confidence intervals, effect sizes, degrees of freedom and P value noted<br>*Give P values as exact values whenever suitable.*
☒	☐	For Bayesian analysis, information on the choice of priors and Markov chain Monte Carlo settings
☐	☒	For hierarchical and complex designs, identification of the appropriate level for tests and full reporting of outcomes
☐	☒	Estimates of effect sizes (e.g. Cohen's d, Pearson's r), indicating how they were calculated

Our web collection on statistics for biologists contains articles on many of the points above.

Software and code

Policy information about availability of computer code

Data collection	Fecal samples for microbiome analysis were collected using the OMNIgene-GUT stool/feces collection kit (OMR-200, DNA Genotek, Ottawa, Canada) and 16S ribosomal RNA sequencing was performed in 599 men by targeting and amplifying the V4 region of the 16S rRNA gene using the 515F and 806R primers. Raw V4 sequence reads were demultiplexed using Illumina's bcl2fastq software version 2.20.0.433. Primers were trimmed via cutadapt 1.18. Primary feature table was generated using Deblur 1.1.0. Phylogenetic tree was built by using SEPP 4.3.5 with Greengenes 13.8. All custom code can be accessed at https://github.com/knightlab-analyses/vitamin-d. DOI: 10.5281/zenodo.4123576
Data analysis	Analysis of microbiome communities was conducted in QIIME 2 (Quantitative Insights Into Microbial Ecology) (2020.2 distribution) and covariate analysis was carried out using R 3.6.1 and Python 3.6.10.

For manuscripts utilizing custom algorithms or software that are central to the research but not yet described in published literature, software must be made available to editors and reviewers. We strongly encourage code deposition in a community repository (e.g. GitHub). See the Nature Research guidelines for submitting code & software for further information.

Data

Policy information about availability of data

All manuscripts must include a data availability statement. This statement should provide the following information, where applicable:

- Accession codes, unique identifiers, or web links for publicly available datasets
- A list of figures that have associated raw data
- A description of any restrictions on data availability

The Knight Lab received the microbial sequencing data in the form of FASTQ files from the San Francisco Coordinating Center. The sequences and biom datasets were submitted to the European Bioinformatics Institute (EBI) database in April 2018, with accession number ERP107984 and are publicly available. All other data, including clinical data, are available from the corresponding author upon reasonable request (email: dkado@ucsd.edu). The raw metabolomic data for the clinical

measures of vitamin D metabolites are not available as they were performed in a large volume clinical laboratory in Leuven, Belgium where the mass spectrometry data are not routinely kept. However, the source vitamin D data files and all other clinical data are stored in the MrOS data online repository. Participant-level personally identifiable data are protected under the Health Insurance Portability and Accountability Act of 1996 (HIPAA), Public Law 104-1919 that mandated the adoption of Federal protections for individually identifiable health information. Thus public distribution is not allowable, but all study data can be made available as a Limited Data Set through accessing https://mrosonline.ucsf.edu. Interested users can create an account by registering online and signing a Data Use Agreement (DUA). The DUA stipulates that the data recipient agrees not to Use or Disclose the Limited Data Set for any purpose other than Permitted Uses and Disclosures or as Required by Law. The full DUA is available here: https://mrosonline.ucsf.edu/Account/UserAgreement.

Field-specific reporting

Please select the one below that is the best fit for your research. If you are not sure, read the appropriate sections before making your selection.

☒ Life sciences ☐ Behavioural & social sciences ☐ Ecological, evolutionary & environmental sciences

For a reference copy of the document with all sections, see nature.com/documents/nr-reporting-summary-flat.pdf

Life sciences study design

All studies must disclose on these points even when the disclosure is negative.

Sample size	567 participants from the MrOS cohort provided matched blood and stool samples for analysis. Our microbiome substudy of the Mr Os cohort, comprised a secondary analysis, and we did not conduct an a priori sample size calculation. With the sample size of 567 older men with gut microbiome samples, we are well-powered to detect meaningful effect sizes as observed in our estimated associations between the gut microbiome(i.e., microbial alpha diversity, beta diversity and specific taxa), and vitamin D metabolites.
Data exclusions	Of 982 men who agreed to provide stool samples, the first 599 stool specimens collected were sent for microbiome analysis in 2016. Of these 599 men, 567 had adequate blood samples for vitamin D metabolite quantification. 32 participants were excluded because they provided stool samples but no associated blood sample for vitamin D quantification.
Replication	Mass spectrometry validation/repetitions for the vitamin D measures were carried out with inter-assay coefficients of variation detailed in the methods section. Briefly, to address variation during mass spectrometry replication, this study included a calibration in each run, the use of isotopic internal standards, statistical process control (repeat serum in each run that has to remain between preset limits before accepting batches), and external quality control schemes (DEQAS (4 times 5 samples every year)). In the paper, we discuss the number of batches (n=7 for 25(OH)D; n=57 for 1,25(OH)2D and 24,25(OH)2D), the calibration procedure (in every batch) and quality control procedure (repeat sera in every batch kept between preset limits) as well as the QC data (between-run imprecision on relevant concentration level of repeat serum: 6.7% at 40 pg/mL for 1α,25(OH)2D3 and 7.6% at 2.0 ng/mL for 24,25(OH)2D3; 5.6% at 28.9 ng/mL 25(OH)D; the median concentrations of 1α,25(OH)2D3, 24,25(OH)2D3 and 20(OH)D in the samples of this study were 56 pg/mL, 3.2 ng/mL, and 34.2 ng/mL, respectively) and the external quality assessment scheme (DEQAS) in which the laboratory participates (with acceptable results).<br><br>The microbiome stool samples were processed using 16s ribosomal RNA gene amplicon sequencing, following the EMP protocol (Caporaso et al. 2012, Thompson et al. 2017). Samples were amplified in triplicate, meaning each sample as amplified in 3 replicate 25-μL PCR reactions and pooled. To ensure replication in data processing and analysis, we used Qiita for primer trimming and quality control using Deblur 1.1.0 and SEPP 4.3.5, and QIIME 2 for analysis of alpha diversity, beta diversity and taxonomy assignment. Jupyter notebooks of R and Python code are provided on github repo: https://github.com/knightlab-analyses/vitamin-d to enhance reproducibility.
Randomization	This manuscript describes a cross sectional study of 567 older men, followed in outpatient clinical study sites without randomization into intervention or control groups. Randomization is not relevant to this study, because this is an observational study by design and no interventions were done. In our analysis, covariates are adjusted using forward stepwise redundancy analysis and multiple linear regression with stepwise backwards selection.
Blinding	As above, this is a cross sectional study without randomization or blinding. This is an observational study rather than an interventional study. As such, there was no allocation step.

Reporting for specific materials, systems and methods

We require information from authors about some types of materials, experimental systems and methods used in many studies. Here, indicate whether each material, system or method listed is relevant to your study. If you are not sure if a list item applies to your research, read the appropriate section before selecting a response.

Materials & experimental systems

n/a	Involved in the study
☒	☐ Antibodies
☒	☐ Eukaryotic cell lines
☒	☐ Palaeontology and archaeology
☒	☐ Animals and other organisms
☐	☒ Human research participants
☒	☐ Clinical data
☒	☐ Dual use research of concern

Methods

n/a	Involved in the study
☒	☐ ChIP-seq
☒	☐ Flow cytometry
☒	☐ MRI-based neuroimaging

Human research participants

Policy information about studies involving human research participants

Population characteristics	Originating in 2000-2002, the Osteoporotic Fractures in Men Study (MrOS) recruited 5994 men aged 65 years and older from six U.S. clinical sites, with continued follow-up involving four full in-clinic examinations through 2016. The six clinical sites were Birmingham, AL; Minneapolis, MN; Palo Alto, CA; Monongahela Valley near Pittsburgh, PA; Portland, OR; and San Diego, CA.
Recruitment	Participants were men enrolled in the Osteoporotic Fractures in Men (MrOS) Study. Briefly, MrOS study participants comprise community dwelling, ambulatory men aged 65 years or older. The inclusion criteria for the study cohort were: (1) ability to walk without the assistance of another person, (2) absence of bilateral hip replacements, (3) ability to provide self-reported data, (4) residence near a clinical site for the duration of the study, (5) absence of a medical condition that (in the judgment of the investigator) would result in imminent death, and (6) ability to understand and sign an informed consent. To enroll, participants had to provide written informed consent, complete the self-administered questionnaire (SAQ), attend clinic visits, and complete anthropometric, DXA, and vertebral X-ray procedures. No other exclusion criteria were used. Recruitment was coordinated by the MrOS Steering Committee, and participants were identified by mailing invitations to men living in the communities surrounding the clinical sites. Men 65 years who resided in the 6 communities were identified from voter registrations, motor vehicle registrations, and HICFA listings. Mailing recruitment efforts were supplemented at some sites with community and senior newspaper advertisements, and presentations to community groups. Each of the 6 participating sites designed and customized these local strategies to enhance the recruitment of minority groups. Surviving participants from the ongoing longitudinal cohort study were invited to provide blood and stool samples between 2015 and 2016. Of these, 567 provided appropriate samples for vitamin D quantification and stool microbiome analysis. Potential biases include survival bias, selection bias for those with registered mailing addresses and those healthy enough to submit a stool sample, and recall bias for survey elements including the food frequency questionnaire. These biases are applicable to older, healthy, community dwelling older white men.
Ethics oversight	The institutional review boards of the six participating institutions approved the study protocol, and written informed consent was obtained from all participants. Ethics approval was provided by each of the Institutional Review Boards of the University of Alabama at Birmingham, University of Minnesota, Stanford University, University of Pittsburgh, Oregon Health and Science University, and University of California, San Diego. Additional oversight was provided by the MrOS Steering Committee, composed of principal investigators from the clinical sites, investigators at the Administrative and Coordinating Centers, other key investigators and representatives from NIAMS and NIA.

Note that full information on the approval of the study protocol must also be provided in the manuscript.
